# Replication and the Establishment of Scientific Truth

**DOI:** 10.3389/fpsyg.2020.02183

**Published:** 2020-09-16

**Authors:** Seppo E. Iso-Ahola

**Affiliations:** Department of Kinesiology, School of Public Health, University of Maryland, College Park, College Park, MD, United States

**Keywords:** replication, reproducibility, scientific truth, scientific method, science, data

## Abstract

The idea of replication is based on the premise that there are empirical regularities or universal laws to be replicated and verified, and the scientific method is adequate for doing it. Scientific truth, however, is not absolute but relative to time, context, and the method used. Time and context are inextricably intertwined in that time (e.g., Christmas Day vs. New Year’s Day) creates different contexts for behaviors and contexts create different experiences of time, rendering psychological phenomena inherently variable. This means that internal and external conditions fluctuate and are different in a replication study vs. the original. Thus, a replication experiment is just another empirical investigation in an ongoing effort to establish scientific truth. Neither the original nor a replication is the final arbiter of whether or not something exists. Discovered patterns need not be permanent laws of human behavior proven by the pinpoint statistical verification through replication. To move forward, *phenomenon replications* are needed to investigate phenomena in different ways, forms, contexts, and times. Such investigations look at phenomena not just in terms the magnitude of their effects but also by their frequency, duration, and intensity in labs and real life. They will also shed light on the extent to which lab manipulations may make many phenomena subjectively conscious events and effects (e.g., causal attributions) when they are nonconsciously experienced in real life, or vice versa. As scientific knowledge in physics is temporary and incomplete, should it be any surprise that science can only provide “temporary winners” for psychological knowledge of human behavior?

## Introduction

This paper examines the nature of scientific and psychological truth and the role of replication in its establishment. It becomes evident from this examination that replication is only a part of the scientific method and does not have any special status in it. In fact, a so-called exact replication is just one type of replication or, at best, an approximation of the original study, and more generally, it is just another type of empirical study. “There are no critical tests of theories, and there are no objectively decisive replications” (Earp and Trafimow, 2015); no such thing as an exact or “direct” replication exists (Stroebe and Strack, 2014; Anderson et al., 2016; Rubin, 2019). Attempted exact replications cannot therefore become the final arbiters of truth any more than the original studies. In essence, then, every replication becomes a “constructive” (Lykken, 1968) or “conceptual” (Crandall and Sherman, 2015) replication that may or may not add to the existing knowledge. Importantly, replications cannot provide yes-no answers to whether or not something exists, even though many argue that “direct replications test the basic existence of phenomena” (LeBel et al., 2017).

Exact or direct replications cannot be the final arbiters of scientific truth because (1) it is impossible to create conditions identical to the original test, and thus failures to replicate methods lead to failures to replicate results; (2) psychological phenomena are not limited to one specific form and condition, but are found in many different situations, and they are inherently subtle and variable due to the effects of time and context; (3) all effects are “interaction effects” even if laboratories are testing “main effects,” leading to a difference between lab truth and real-life truth; and (4) the methods are psychometrically inadequate for giving categorical answers due to the problems of unreliability, invalidity, and sampling errors.

All attempts at exact replications are doomed to fail not because the investigated psychological phenomenon is not robust enough to reveal itself repeatedly, but because they attempt to replicate something whose existence is not limited to a specific context and specific time. By definition, exact replications rest on the assumption that a phenomenon exists only in the condition identical to the original, but overlook the reality that the tested phenomenon can exist in various forms and under different conditions. As the null hypothesis cannot be confirmed by non-existing exact replications, it is not surprising that such replication attempts have failed; logically, in fact, it is surprising that all *method replications* have not failed because they should have. Undoubtedly, some replications have failed because original studies were conducted under the “old rules” by not following today’s stricter guidelines [e.g., p-hacking (Simmons et al., 2011)]. But focusing on past failures of method replications loses sight of the main thing: phenomenon and its boundary conditions.

In contrast, *phenomenon replications* test phenomena in varied forms, contexts, and times using different methods and consequently provide more nuanced and refined explanations than categorical declarations that the phenomenon is or is not real (Doyen et al., 2012; Carter et al., 2015; Gerber et al., 2016). They examine effects using multiple criteria other than the magnitude, such as frequency, duration, and intensity, as well as factors that give rise to phenomena and those that reduce their influence. They also shed light on boundary conditions and theories’ strengths and weaknesses, thereby helping modify and expand theories. Phenomenon replications are constructive in nature but are not limited to single replications. Rather, they are programs of ongoing studies examining phenomena in different forms, contexts, and times.

Wegner (1994) presents good examples of phenomenon replications. He tested the same phenomenon (making an ironic error of mental control by actively avoiding thoughts of an object or action under mental load) employing different cognitive and behavioral tasks and contexts. Obviously, his methods and manipulations were different in different experimental situations, but they nevertheless reproduced the same result and additively and informatively showed that the effect is more pronounced in some tasks and situations (e.g., thought suppression) than others.

In a similar vein, Milgram’s way of experimentally investigating obedience is just one of many ways of studying it. Although Milgram’s findings have been replicated (Burger, 2009; Doliński et al., 2017), failures to directly replicate his original findings likely reflect methodological modifications, not necessarily that the phenomenon fails to influence outcomes (Elms, 2009). Moreover, experimental and non-experimental methods vary considerably in their sensitivity and ability to unveil phenomena. Since the same psychological phenomenon appears in different forms and to different degrees in varied contexts, a diversity of methodological approaches is necessary for replications of the originals findings. The accumulating, convergent evidence will provide a better understanding of possible false positive and false negative results of the original findings and, thus, of the nature of the investigated phenomenon.

Due to the fundamental limitation of the empirical method, no permanent scientific truth (or its absence) can be established by empirical replications. Phenomenon replications, however, can provide useful data and information and thereby improve estimates of the effects. But like original tests, replications can produce both false positives and false negatives, as these determinations are based on strict and arbitrary criteria, predominantly statistical thresholds (previously *p*-value, now Effect Size, Confidence Intervals, and Bayes Factor). A danger is that the search for underlying causes becomes largely a “statistical exercise” (Grice, 2014), even though “a statistical procedure is not an automatic, mechanical truth-generating machine for producing or verifying substantive causal theories” (Meehl, 1992, p. 143).

Statistical determination of scientific truth is based on the assumption that psychological attributes and phenomena are quantitative, but are they (Sherry, 2011; Grice, 2014)? If they are quantitative, a problem then becomes one of an agreement about the level at which psychological phenomena can be declared genuine and real. However, the agreement is not only about the quantity but more importantly, the meaning of numbers assumed to represent psychological constructs. This question of construct validity (Cronbach and Meehl, 1955) poses major challenges for psychological research in general and replications in particular, as “replicability does not equal validity” (Hussey and Hughes, 2020).

Taken together, however difficult or insurmountable the empirical testing of it would be, aliens’ existence cannot be discounted, and so-called failed replications cannot declare any hypothesis logically or theoretically invalid. Similar to replications in physics, replications in psychology can only speak to observations about affect, cognition, and behavior in a specific context at a specific time. But this does not obviate the discovery of patterns that hold for certain situations and times (Lykken, 1991). In general, however, replications are logically tenable only if psychological phenomena can be claimed to be fixed and permanent entities, stable particles that can be described by absolute quantities. In the absence of well-founded claims, the basic premise of replication can be questioned.

## Nature of Scientific Truth

Implicit in the idea of empirical science is a question: What is truth anyway, and how is it determined? Replication studies attempt to answer this question by seeking to show if the original finding can be obtained again under similar experimental conditions. In science, truth, whatever its content, is said to exist to the extent that it is theoretically and empirically supported. Accordingly, a successful replication of an original finding is taken to mean that a truth exists, while a failure to replicate supposedly indicates the absence of the presumed truth. This fundamental axiom rests on the assumption that a scientific truth exists in the first place, and that it would reveal itself on researchers’ empirical demands again and again. However, if an effect does not respond to replicators’ call, the weight of evidence shifts against it or, worse, its existence is cast in doubt and void, as has recently been done with regard to ego depletion, social priming, bystander effect, actor-observer asymmetry in attributions, loss aversion, delay of gratification, and other phenomena (Malle, 2006; Doyen et al., 2012; Carter et al., 2015; Gerber et al., 2016).

Whether a phenomenon has truly revealed itself is decided by statistical means, typically *p*-value. It can work in physics (Meehl, 1967) where, for example, multiple experiments in Switzerland revealed the odds of one in 3.5 million in favor of the existence of the Higgs boson particle (or that the result would occur if the null hypothesis were true). In psychological studies of human behavior, however, effects of such magnitude and precision do not exist, as the same experimental treatment can produce a *p*-value of 0.001 today but 0.75 tomorrow (Cumming, 2014).

Besides the statistical problem, the yes-or-no determination is logically untenable because the absence of evidence derived from a replication is taken to indicate that a phenomenon does not exist (Carter et al., 2015; LeBel et al., 2017). The absence of evidence can result from many constraining factors, methodological and measurement factors on one hand and time- and context-related determinants on the other, and cannot therefore be taken as evidence for the absence of a truth or phenomenon (Trafimow, 2003). Moreover, because empirical support is always provisional and propositional, and therefore preliminary, conditional, and relative, the categorical determination of scientific truth is not possible. Accumulating evidence from phenomenon replications, however, provides a better understanding of the phenomenon and its temporary truth-value.

The preponderance of evidence for a phenomenon only provides a more probable or justified explanation than other explanations at the present time (Kuhn, 1962; Meehl, 1990), but offers no final truth (McFall, 1996). A current theory or explanation has not yet been shown false or has not been disproven or “falsified” (Popper, 1959), nor have alternative hypotheses been accepted by “strong inference” (Platt, 1964); for a good example of strong inference ruling out alternative explanations in empirical research, see Oppezzo and Schwartz (2014).

## Measurement and Replication

In science, the nature and acceptance of truth is importantly shaped by measurement. This means that replications are essentially about measurement invariance and reliability of previous findings, especially if studies aim at exact replications. In principle, if a phenomenon is successfully replicated, it is viewed as a *reliable* and bona fide effect, and a scientific truth, albeit temporary, is therefore established. However, measurement invariance is not just a matter of reliability, but validity as well (Hussey and Hughes, 2020). That is, does a successful replication automatically capture the underlying mechanism of the investigated phenomenon? Was the underlying or latent construct measured validly?

While reliability is important, a more critical issue is validity. An original finding can be replicable but nevertheless lead to invalid conclusions, because replicability is not the same as validity (Hussey and Hughes, 2020). If we can measure something reliably but it is off the target, such a replicated finding means little for understanding the underlying phenomenon. In other words, what does a successful or unsuccessful replication with an invalid measurement or test mean for scientific truth? If, for example, behavioral tasks are used as dependent measures to capture the mechanism underlying self-regulation, such a research strategy is problematic because behavioral tasks have been shown to possess a relatively low test-retest reliability (Enkavi et al., 2019). As reliability and validity are interrelated, lower reliability leads to lower validity, meaning that behavioral measures are less accurate and valid for measuring the underlying mechanism for self-regulation (Enkavi et al., 2019). Low reliability, and thus low validity, of behavioral measures increase replication failures.

The problem of validity is also a matter of poorly defined theoretical constructs, resulting in a problem of overlapping constructs and measurement variance (Hanfstingl, 2019). For example, there is considerable theoretical overlapping between such constructs as “grit,” resilience, self-control, mental toughness, and “self-as-doer.” In a similar vein, a single term or construct can have different meanings. Smiling as a response to a stimulus may be replicated reliably, but it often has different meanings in different contexts.

Given that psychological scientists seek to provide robust explanations for various phenomena through latent constructs, measurement validity becomes a critical issue (Hanfstingl, 2019). Thus, replicable findings are useful only if they reflect differences in latent variables, not just how reliably participants interpret items in the questionnaire (Hussey and Hughes, 2020). From the validity standpoint, then, experimental participants’ performance should be driven by the underlying construct, such as physical fitness being the construct determining the treadmill test performance (Secrest, 1984). A resultant number has a specific meaning that represents the underlying construct, reflecting its construct validity (Cronbach and Meehl, 1955). In this common quantitative approach, validity is conceptualized as a matter of degree, not as qualitative concept of “yes” or “no” (Cronbach and Meehl, 1955; Messick, 1989).

Although it is often thought that reliability sets the upper bound for validity (Lord and Novick, 1968), technically speaking, this is inaccurate because the maximum validity of a test is the square root of the reliability (Secrest, 1984). Nevertheless, increasing measurements’ reliability in original and replication studies is necessary; unfortunately, it is not uncommon to find reported reliability coefficients to be less than the recommended standard of 0.70 (Nunnally, 1978). For example, in task-fMRI studies, measures’ average test-retest reliability (0.397) is very poor, making them unsuitable for brain-behavior mapping for both research and clinical purposes (Elliott et al., 2020). In sum, successful or unsuccessful replications based on highly reliable and highly valid measurements will appreciably add to the existing knowledge. If, on the other hand, the original study and follow-up replications were based on relatively unreliable and invalid measures, such studies would have little, or no, value in the establishment of scientific knowledge and truth.

Even in physics the present scientific truth is subject to revisions due to measurement problems and use of different methods. For example, two groups of scientists have arrived at vastly different numerical values for the rate of the expansion of the universe, with one indicating that the universe is receding about 9% faster than the other (Panek, 2020). A great puzzle among astrophysicists is whether the discrepancy is due to a systematic error in one of the two measurements or whether a “new physics” (e.g., dark energy changing over time) altogether is needed to explain the “inflation” of the universe. In a similar vein, the beginning of the universe itself has been questioned by some physicists, who have suggested that Big Bang may not be taken as an unchangeable conclusion and truth; accordingly, Big Bang could actually be “Big Bounce” in a constellation of infinite universes (“multiverses”).

By the same token, there is no denying that certain laws of the nature are permanent and fixed entities. For example, light always travels at the constant speed and faster than anything else in a vacuum, even if it slows down to 75% of the vacuum speed in water. There are, however, no such permanent laws in psychology because psychological phenomena are not fixed and unchangeable particles. Yet stability (e.g., speed-accuracy tradeoff) exists in human behavior. As stable patterns are tendencies, not laws, in human affect, cognition, and behavior, they become less stable under certain conditions. This is a challenge for empirical science in psychology in general and for making generalizations about human behavior in particular.

The above suggests a major difference between physics and psychology. While the laws of nature are fixed and stable entities, though subject to revisions, psychological phenomena are a mixture of both variability and stability. In illustrative terms, when light travels at its constant speed, it does not have an ability to slow down at Jupiter to admire the scenery, whereas the human mind does it in various contexts, processing information differently as a function of internal and external conditions. Because humans are capable of changing and evolving, their feelings, cognitions, and behaviors can fluctuate substantially, but at the same time, there is a considerable degree of stability to them (Hudson et al., 2017). In fact, at the fundamental level, attention and visual search are biased toward temporal regularities (Zhao et al., 2013). The upshot is that the establishment of scientific truth is as hard, if not harder, in psychological as in other sciences. There are no invariant particles to be discovered beyond a shadow of doubt in psychology, but instead, variable, temporary, context-dependent, and subtle phenomena (Iso-Ahola, 2017). As a result, replications can only provide experimental feedback for hypotheses and theories, but not declarations that certain phenomena are not real.

## Lab Truth Versus Real-Life Truth

The replicability problem is evident when considering that the nature of psychological phenomena is intertwined with their methodological/measurement demonstrations. Accordingly, self-control prevents people from using profanities in public (equivalent to lab situations), but not when they interact with trusted friends in private settings (real-life situations). Field experiments, while important for external validity, tend to compromise internal validity (Shadish et al., 2002) and make the findings of such studies difficult to replicate. Because intervening variables are hard to experimentally control in real-life situations, the measured influence of these variables will not be identical in original and replication studies. The tradeoff between internal and external validity is nothing new but should not be ignored, because it suggests a marked difference between lab truth and real-life truth.

In theory, phenomena exist in lab settings but not in real life, and vice versa. This yields four scenarios according to where replications have been conducted and whether replications have succeeded or failed: (1) successful replication in lab, (2) successful replication in real life, (3) unsuccessful replication in lab, and (4) unsuccessful replication in real life. Following the first case, a question arises: Is this lab phenomenon a bona fide effect in real life as well or just limited to lab situations as a methodological artifact? Although reaction time, and its determinants, can reliably be replicated in labs, it may be less replicable in real-life situations (e.g., a slowed reaction time in traffic due to fear). Similarly, causal attributions can be elicited in labs by asking participants to causally attribute their performance outcomes, but in real life, people rarely engage such conscious thoughts.

Regarding the second situation, a question is whether these successful real-life replications make the phenomenon more credible, as in the case of replicated social priming effects in a variety of real-life situations with real-life variables (Bargh, 2014), even if some lab experiments have failed to replicate them. It could be argued that the greater the number of real-life situations in which the phenomenon has been replicated, the greater the likelihood that it is a bona fide effect, especially if the empirical demonstrations are consistent with lab replications. In general, empirical demonstrations and replications in both lab and real-life settings provide the strongest evidential support for phenomena, whereas a lack of both lab (situation 3) and field confirmation (situation 4) provides the least support.

In the third case, the phenomenon does not exist as a lab effect but raises questions: Does the absence of lab evidence rule out methodological artifacts as the reason for replication failures, and do these failed lab replications necessarily rule out the phenomenon’s viability in real life? Ego depletion may a weak phenomenon when participants perform behavioral lab tasks, but it could be a strong effect in everyday life contexts (Iso-Ahola, 2017). In the fourth situation, evidence is lacking in both labs and field settings and therefore suggests an end to scientific inquiry on the phenomenon. However, it is possible that methodological problems and artifacts have failed to reveal the phenomenon (e.g., cognitive dissonance) in real-life situations.

The four scenarios suggest a complex and non-categorical role for replication in psychological science. Scientific truth is stronger when it is based on corroborated evidence obtained from lab and real-life situations. However, some have described psychology as the science of self-reports and finger movements and have asked, whatever happened to actual behaviors (Baumeister et al., 2007)? It is therefore not surprising that cognitive neuroscientists, for example, have recently called on researchers to investigate the relationship between the brain and human behavior in the context of everyday experiences (Shamay-Tsoory and Mendelsohn, 2019). Such “ecologically valid” studies are to be based on “representative designs” in which both individuals and stimulus situations are representatively sampled for better understanding and generalizability (Brunswik, 1955). Real-life human behavior is always dynamic, interactive and complex, and therefore poses major challenges for controlled experimentation. Nevertheless, real-life replications are just as important as lab replications for providing cumulative and convergent evidence for various phenomena.

## Stability and Variability

That a need for the balance between stability and variability is deeply embedded in human nature and manifested in behavior is well documented. For example, college students spend about half of their time doing the same things day after day, but the flip side is that they spend the other half doing different things (Wood et al., 2002). Thus, they seek both stability and variability, consistency and novelty, in their lives. According to Berlyne (1960), this tendency is driven by the need for optimal arousal. The need for variety (and stimulation) is so great that experimental participants find sensory deprivation conditions intolerable, as illustrated by their request to hear a recording of an old stock market report over and over (Murray, 1964). This raises a question for experimental research: Is participants’ need for variability and stability met to the same degree from one study to another? Are participants under- or overstimulated to the same extent?

Overall, both emotions and cognitions have stability and variability to them, be they implicit or explicit attitudes (Charlesworth and Banaji, 2019), implicit biases (Vuletich and Payne, 2019), or day-to-day affect (Hudson et al., 2017). Recent evidence further indicates that affect variability, not just the mean stability of positive affect, is associated with health-related physiological outcomes (e.g., antibody response to vaccination) (Pressman and Cross, 2018). In short, it is not surprising why replications of certain psychological phenomena have failed; they have in part failed because it has been assumed that participants are affectively and cognitively stable from experiment to experiment and their need for variability and novelty does not influence their experimental performance. Without measuring these constructs and their proxy variables, it is not possible to determine the extent to which the basic need for stability and variability contributes to differences between experimental findings.

The inherent variability of psychological phenomena can further be influenced by contextual and time-related factors (Ramscar et al., 2015; Bavel et al., 2016; Ramscar, 2016). Every experimental situation is a different social context and can interactively with time affect participants’ behavior. Psychological phenomena do not occur in frozen time and context but vary substantially as a function of these determinants. Human behavior is dynamic, continuous, and interactive, even if it is studied in frozen contexts and at frozen times in research labs. Furthermore, even if people tend to think and act non-consciously most of the time (Bargh, 1997; Dijksterhuis and Aarts, 2010), they have cognitive capacity to change their feelings and thoughts in any place at any time (Baumeister et al., 2011; Dehaene, 2014). Experimental settings and instructions could easily give rise to such changes (Belletier et al., 2019), which suggests that it is imperative to follow verbatim experimental instructions and scripts in replication studies (and include them in publications). Phenomenon replications are therefore needed to examine the effects of deviations from and variations in experimental details on replication results.

In efforts to create equally homogeneous external conditions, original studies and their replications seek to constrain lab conditions to the point where experimental participants are stripped of their very psychological being and behavior. Yet, in everyday life, psychologically functioning individuals have freedom to feel, think, and act as they desire in specific situations at specific times, thereby creating complex interactive relationships. These internal conditions, however, cannot be made entirely or even sufficiently homogeneous from one lab to another and yet, it is these internal conditions in conjunction with external factors that determine variability in participants’ responses. They (e.g., conscious thoughts) can be suppressed by strict and artificial methodological requirements, but it is unclear whether such suppression is the same between the studies.

Thus, it is not known if the replication participants have had identical conscious feelings and thoughts of excitement or boredom, for example, to say nothing about non-conscious feelings and thoughts. In the reported replications, no attempt has been made to create internal conditions homogeneous relative to the original conditions. There is not a single replication study reported that would have shown participants’ feelings and cognitions—both conscious and non-conscious—to be precisely the same with those of the original participants. Although people generally are inclined to rely on non-conscious processing of their thoughts, emotions, and behaviors (Kahneman, 2011; Bargh, 2017), they can deviate from this tendency at any time (Baumeister et al., 2011), and experimental instructions can produce different degrees and ratios of conscious vs. non-conscious processing from one study to another.

These degrees and ratios, however, can be estimated and should be taken into account to separate real effects from extraneous influences. It is proposed, on the basis of a vast literature on conscious vs. non-conscious processing (Kahneman, 2011; Bargh, 2017), that the greater the degree of conscious processing required for performing an experimental task, the less replicable are the findings because of increased within-person and between-person variance; and in reverse, the greater the role of non-conscious processing, the more replicable are the results.

It should be noted that some experimental tasks (e.g., a right-handed person doing something with his/her left hand) require greater cognitive awareness and resources than others, and that most tasks become increasingly non-consciously processed with repeats (Bargh, 2017), thereby diminishing cognitive fatigue (Hockey, 2011). This suggests that a replication may have failed because the experimental task, instructions, and manipulations led participants to engage in different degrees of conscious vs. non-conscious processing and use of cognitive resources or because the phenomenon is inherently associated with one type of processing vs. the other. For example, do people get more ego-depleted when doing consciousness-demanding vs. non-consciousness-demanding tasks?

The complexity of the human mind means that psychological phenomena are essentially interaction effects (Mischel, 1973), products of time and social context, thereby increasing the likelihood of failed replications. Both the beauty and challenge of the human mind for researchers is that people’s thinking varies from simple to complex, from what might be called simple “main-effect thinking” to complex “interaction-effect thinking” (Iso-Ahola, 2017). In their thinking (conscious or non-conscious), people can be simple at one time and complex at another, or both at the same time. They can be either rational or irrational or both in the same situation (Tversky and Kahneman, 1971; Lerner et al., 2004; Lieder et al., 2018).

How does a replication researcher know the mode of thinking in which his/her participants are vis-à-vis the participants of the original study? In short, it cannot be assumed that experimental instructions will make every participant think, both consciously and non-consciously, in the same way. This emphasizes the necessity of reporting precise experimental instructions in publications. Such information would allow researchers to identify within- and between-subjects factors that cause individuals to react differently to instructions.

The complexity of psychological phenomena means that many variables will interactively influence outcome variables, which poses major problems for replications. As a whole, other factors being equal, the more complex and interactive the effects, the less replicable and reproducible they are. Meehl (1990) famously concluded that “everything is correlated with everything, more or less.” An important implication is that the manipulation of a focal independent variable influences other causal independent variables even in randomized experiments, and these other independent variables either reduce or increase the effect of the focal variable. Such interaction effects can invoke divergent thinking in participants and thus lead to more variability in behavior from time to time, from situation to situation, which in turn reduces the probability of successful replications of the original findings, as well as predictability of behavior.

## Temporary Truth

Whether pursued through inductive or deductive reasoning and methods, scientific knowledge is subject to continuous revisions, resulting in temporary and preliminary truth. Since investigated effects are time-bound, there are only “temporary” winners in knowledge and no final truths (McFall, 1996). A good example of the temporary nature of psychological knowledge can be seen in the virtuous cycle of daily motivation. As people’s senses of strivings are dynamic, they change from day to day so that today’s need satisfaction at work can enhance the next morning’s strivings and work behaviors (Foulk et al., 2019). How, then, can replication studies be expected to reproduce the original participants’ feelings, thoughts, and acts when they change from one working day to another? Can it be assumed that these factors vary, similarly, from study to study, especially when participants are typically not randomly selected? What is the underlying pattern here to be replicated? Is it that people are dynamic, changing individuals, or is it just motivation?

If replications fail to reproduce the original findings, it could easily be because of the dynamic cycle of daily motivation and the measurements taken at different times of the day. Related to this point, the effect of coffee on cognitive performance may depend on the time of drinking (morning vs. afternoon) (Ryan et al., 2002), and the effects of verbal cues on voting behavior are contingent on whether measurements are taken 1–2 days vs. 3 days prior to actual voting (Bryan et al., 2011; Gerber et al., 2016). All of this again underscores that humans are complex and variable beings, and this complexity and variability therefore needs to be accounted for theoretically with parsimonious models (Saylors and Trafimow, 2020), and empirically with high-powered studies in labs and real life.

Another thing to be noted is that psychological effects occur both in short and long periods of time. In the latter, the effects often become reciprocal, as demonstrated in the reciprocity of prosocial behavior and positive affect in daily life (Snipper et al., 2018), reciprocity of self-control depletion and self-control practice (Baumeister, 2015), reciprocity of athletic performance and the perceived size of softball (Witt et al., 2012), and reciprocity of self-concept and athletic performance in a specific performance situation (time) and cumulatively over time (Marsh and Perry, 2005). Thus, a replication experiment may fail to reproduce an original finding because it is a frozen snapshot of one-way effect in time when the effect actually fluctuates with time due to a continuous reciprocal relationship between the variables. In a game situation, for example, performance continuously affects the perceived size of the ball, which in turn continuously affects performance (Witt et al., 2012). If a field experiment is replicating the former relationship but ignores the latter relationship, the replication is likely to fail. For this reason alone, it is not logically possible to declare that a phenomenon is permanent or that it does not exist.

It should also be noted that although one-time lab exposure to a stimulus (e.g., meditation training) can produce an observable effect, long-term exposures are more likely to result in more replicable effects because they solidify and reinforce single-exposure or training effects (Kok et al., 2013). In medical science, for example, the establishment of the efficacy of a treatment often requires long-term exposures to drugs. Similarly, environmental toxins may take years to produce harmful effects (e.g., smoking and lung cancer). Even if the findings of the original study were obtained from a short-term exposure to a specific toxin, the effect is more likely to be replicated following repeated exposures to it. However, there is no guarantee that a toxin as a single causal factor will lead to successful replications. As noted earlier, the manipulation of a focal independent variable can influence other causal independent variables.

In general, the same principle applies to replication of psychological effects. Because human behaviors are products of multiple time-bound and context-bound factors, and are therefore products of interaction effects, psychological phenomena can only increase the probability of the occurrence of certain responses and actions. Thus, a psychological effect does not itself cause changes in a behavior but interactively contributes to them. For example, it is known that social priming produces differing motivational effects rather than a single default effect (Loersch and Payne, 2011). The absence of such one-time generalizable effects inevitably reduces the replicability of original findings. The situation calls for better and more accurate theories and models.

Finally, time could play a different role depending on the degree of proximity to major social and religious events and periods. If, for example, original participants’ favorite sport team just lost a championship game, they would likely be in a different mood than replication participants who recently had more pleasant experiences. Is it known that multi-lab and multi-culture replication participants match the original participants on such accounts? In addition to developing better theoretical models to account for this potential variability, methodological and statistical solutions involve large sample sizes, mixed-effect models, and treatment of lab or culture as a factor in replications.

## Context-Bound Truth

Psychological truth is not only time-bound but also context-bound. In general, unlike particles (e.g., photons carrying light), humans do not function and operate in vacuum or optimal experimental conditions but instead in social environments, influencing them and being influenced by them. Contexts may be similar but never identical. Although physical contexts can be reproduced, identical social situations cannot be re-created. For example, spectators go to the same stadium to watch a football game on consecutive Saturdays, but every game is a different social context for many reasons, even though the football game itself is a constant from Saturday to Saturday. Needless to say, no replication can duplicate a previous game’s psychological context, which means that psychological truth is relative to contexts in which a behavior occurs. Since contexts change with time, psychological truth is variable by its nature. How to quantify and predict this variability becomes a key theoretical and empirical issue.

Thus, the same degree of anxiety can cause “choking” (deteriorated performance) in one situation but not in another. Notwithstanding methodological problems, replication failures likely reflect the investigated phenomenon’s inherent variability due to its sensitivity to situational influences (Bavel et al., 2016). This contextual sensitivity also means that instead of making generalized claims, original findings have to be viewed with empirical limitations and uncertainty in mind until the conditions reliably producing certain effects are well understood. What factors make various phenomena more or less sensitive to environmental influences, and thus more variable than stable, remains to be examined both theoretically and empirically.

As time and context are inextricably intertwined, human behavior varies as a function of the interaction of the two, with one affecting another. Contexts vary with time as time creates different contexts, and contexts (e.g., boring vs. exciting lectures) create different experiences of time. Christmas Day is a time that affords a different context and experience than New Year’s Day. With time and context, internal and external conditions change and are therefore different in a replication study when compared to the original.

Time-bound and context-bound effects mean that psychological truth is also person-bound. In other words, psychological truth has intrapersonal boundaries. Intrapersonal differences suggest that the same person is not always similarly affected by time- and context-related variables. There is variability and stability within a single individual’s responses, while interpersonal differences indicate that people differ from one another in the degree to which time and context influence their affect, cognition, and behavior. Psychological studies are predominantly conducted to determine between-subject variance while ignoring within-subject variance. In this approach, intrapersonal (in)consistency gets statistically buried in interpersonal (in)consistency, with weak between-subject averages potentially hiding strong and meaningful intrapersonal effects. However, the problem can in part be remedied by using mixed-effects models to account for between-subjects variance, as well as within-subjects variance by treating stimuli as random effects.

To better understand psychological effects, variability and stability need be investigated and replicated at both intrapersonal and interpersonal levels. In medical research, between-subject determinations (experimental vs. control group) of a new drug’s efficacy may indicate lifespan extension, on average, by only a few months, but individually the effect could be several years for some. Although psychological experiments do not directly deal with life-and-death effects, they need to pay more attention to intrapersonal stability and variability of various effects.

## Subtle Truth

Scientific truth can be subtle and elusive. The first discovery of gravitational waves (2015), as predicted by Einstein’s theory, was more or less accidental and could easily have been missed or misinterpreted. Similarly, a particle presumed to constitute dark matter continues to elude physicists, a situation not uncommon to psychologists. Nevertheless, “a good deal of good physics has been done without high quantitative precision” (Chang, 1995, p. 169). There is no reason why the same could not be said about psychology, even though a notable difference between physics and psychology is that psychological phenomena are not particles that prevail across time and situations. Instead, they vary in time and space and therefore become elusive and subtle, and open for misinterpretations.

Consider, for example, the human face. It can give subtle signs of different emotions as responses to certain stimuli, but those signs can be different in different situations at different times even when exposed to the same stimulus. In laboratories, participants have been able to make reliable judgments from government officials’ faces regarding their likelihood of having been convicted of corruption (Lin et al., 2018). Although such “main effects” may be replicated, interaction effects (i.e., if target persons’ talk and non-verbal behavior are included in the analyses) may not.

Or, consider sarcastic comments. In a subtle way, they can communicate different messages, which can easily be misinterpreted by others. These kinds of subtle effects can lead to the problem of “inferential reproducibility” (Goodman et al., 2016), meaning that different scientists draw different conclusions from the same results. Such inferential errors abound in replication studies of various phenomena, from the effects of subtle linguistic cues (Gerber et al., 2016) to the effects of ability vs. practice on expert performance (Ackerman, 2014; MacNamara et al., 2014). These errors have been particularly common in so-called failures to replicate social priming effects. But as it has been pointed out, associations between priming words and the world are not stable over time because of learning and experiential effects (Ramscar, 2016). It is therefore not surprising that many exact replications of social priming have failed and will fail statistically (Ramscar et al., 2015).

Subtle effects do not necessarily mean that they are not noticeable and important effects. For example, the tone of one’s voice can in a subtle way communicate strong positive or negative feelings to others, yet statistical data analyses could indicate weak effects. Small effect sizes and low multiple correlations can be indicative of important effects, and an unsubstantial manipulation of the independent variable can produce non-trivial effects (Prentice and Miller, 1992). Furthermore, subtle variations in experimental settings from one study to another (e.g., the experimenter’s behavior) “may cause dramatic changes in performance” (Belletier et al., 2019), resulting in conflicting findings and failed replications. Similarly, experimenter beliefs rather than the primed condition can alter participants’ behavior (Gilder and Heerey, 2018). It may therefore be proposed, other things being equal, that the subtler the effects, the greater the likelihood of replication failures; however, such failures cannot necessarily deny the underlying effect. In short, it is clear that the subtlety of psychological effects on one hand and their sensitivity to social contexts on the other pose major problems for replications.

To be sure, some phenomena are manifested in strong and thus potentially more replicable effects, as has been shown by research dealing with the influence of the mere sight of individuals with a gun on subsequent aggressive thoughts (Bushman, 2018). By analogy, vigorous exercise produces stronger physiological effects than moderate and mild exercise. Rarely, however, do people in everyday life function under extreme psychological conditions, nor are the psychological effects likely to be linear, as in the case of exercise intensity. Moreover, the magnitude of an effect is only one criterion by which a phenomenon’s viability can be assessed, and in fact, it may be a poor or inappropriate criterion in many situations. Phenomenon-focused replications would look at an effect not just in terms of its magnitude but also by its frequency, duration, and intensity in labs and real life, as well as whether the phenomenon (e.g., stereotypes) is consciously or non-consciously experienced (Iso-Ahola, 2017). It is known that most psychological phenomena become non-consciously experienced emotions, cognitions, or acts with time (Bargh, 1997, 2017).

Subtle effects may not be quantitatively strong and yet be qualitatively and theoretically meaningful. It has been shown that small effect sizes can potentially be important for both theoretical and practical or utility purposes (Abelson, 1985), as in the case of the effects of mild exercise on cardiovascular health. However, given that psychological studies are statistically underpowered (Rossi, 2013) mainly because of small sample sizes, they are not capable of detecting small effects; large samples are needed to detect small (and subtle) effects. It should be noted that although increasing sample size increases informativeness and power, “high power does not necessarily imply an informative or high-quality experiment” (Cumming, 2014), nor can the average power serve as a “replicability estimate” (McShane et al., 2020).

Although strengthening experimental manipulations increases effect sizes, it can undermine effects’ subtlety. The other side is that because of ethical and moral reasons, some psychological variables cannot experimentally be manipulated to the level at which they occur in real life. As a result, weak lab manipulations are likely to produce weak effects that do not replicate. To counter these problems, phenomena need to be investigated in a diversity of experimental and real-life tasks and situations with different types and degrees of manipulations. In the long run, such constructive replications will yield convergent evidence rather than yes-no determinations of phenomena’s existence.

It would be a mistake to demand that subtle psychological phenomena produce linearly and quantitatively increasing effects before they are deemed real. Such statistical demands would ignore the complex, interactive, non-linear, and subtle nature of psychological phenomena and would reduce them to statistical phenomena. Stringent statistical demands create an illusion of objectivity but do not eliminate subjective questions about “inconsequentially small” but important effects or their “practical meaning,” as has recently been debated regarding the long-term effects of delay of gratification on behavioral outcomes.

Statistical decisions are not error-free either. It has been suggested that some replication failures may be due to applications of the wrong type of Type I error testing procedure: Neyman-Pearson vs. Fisherian Type I error rate (Rubin, 2019). New statistics are no panacea either. Meta-analyses (effect sizes), for example, have led to contradictory findings regarding various phenomena (Hagger et al., 2016; De Vrieze, 2018), although they can be useful in suggesting moderating or interactive effects. In the end, however, statistics cannot yield automatized yes-no decisions on substantive hypotheses’ believability because “there will always remain an important role for expert judgment” (Trafimow, 2019).

In sum, the subtlety of psychological phenomena and their sensitivity to social influences inherently cause variations in empirical observations and pose challenges for reproducibility. There is an important difference between a phenomenon’s subtlety and its observed statistical strength. Feedback, for example, can be given in a subtle or concrete way, but it is only in the latter situation that feedback is likely to show statistical strength. A danger is that a phenomenon’s subtlety will be lost if it has to be manipulated to an artificial statistical strength, turning naturally occurring subtle effects into statistically strong but theoretically questionable effects. A long-term danger is that psychological phenomena will increasingly become defined as statistical phenomena.

## Causality and Truth

The elusive and complex nature of psychological phenomena is reflected in their underlying causes. Let us consider the much-investigated “expert performance” as a phenomenon. Are “talent” (Ackerman, 2014) and “deliberate practice” (Ericsson et al., 1993) necessary or sufficient for exceptional performance? Evidence indicates that each is necessary but not sufficient for producing expert performers in any area of human performance. Most psychological phenomena, however, are not based on yes-or-no necessary causes (e.g., the female gender being necessary for pregnancy), but when they are, it is likely that necessary causes are more replicable than sufficient causes.

Although talent is necessary for expert performance, its contribution varies from one performance domain to another, and thus potentially from one replication to another, depending on how performance is defined and measured (Ackerman, 2014). Even if replications would show talent’s contribution to vary in percentage and be weaker than that of deliberate practice, such replications could not deny the basic fact that talent is necessary for expert performance. Typically, however, psychology experiments seek to establish sufficient causation, as follows:

A. If **X** (self-control resources exhausted during a working day) occurs and **Y** (ego depletion effect) is observed, then the **X-Y** causation is true.
B. If **X** does not occur and no **Y** is observed, then the **X-Y** causation is indeterminable.
C. If **X** occurs but no **Y** is observed, then the **X-Y** causation is untrue.
D. If **X** does not occur but **Y** is observed, **Y** is true but independent of **X**; no **X-Y** causation.

In the above scenarios, A and B, when taken together, seek to establish sufficient causation, in that when **X** is present it produces **Y**, but when it is absent **Y** does not occur. This is the usual treatment-control group situation in experimental research, but it is only one-time demonstration of sufficient causation. Further, an experimental confirmation of the effect of **X** on **Y** would not establish the necessary causation, according to which **X** *must* be present for the effect to follow. It is possible that **X** is necessary for **Y** to occur, but a researcher does not know it from one experiment; a series of experiments would have to substantiate it. And, if the D scenario is encountered, it would deal the death blow to the idea of necessary causation. Using the above example, the ego depletion effect (self-control failure) was observed not because of **X** but because of other factors, such as physical or mental tiredness or habit-supporting cues (e.g., a remote control prompting TV watching). These other factors can empirically be shown to be sufficient causes for **Y**’s existence.

Finally, the C scenario would represent a failed replication of the effect of **X**, suggesting a lack of sufficient causation that whenever **X** is observed it will lead to an effect. Given that psychological effects fluctuate with time and context as a function of internal and external conditions, the refutation of sufficient causation by replication becomes untenable in logic and reality. Furthermore, if there is not a single cause whose presence will always lead to the effect, the entire premise of sufficient causation is in question. In general, the difficulty of empirical verification (replication) of causation stems from the fact that there are no single causes that are both absolutely necessary *and* absolutely sufficient (for a more detailed discussion of probabilistic causation regarding intervention effects and “the probability of sufficiency”, see Pearl and Mackenzie, 2019).

To further elucidate the problem of demonstrating necessary and sufficient causation in psychological research, let us consider research on delay of gratification. Is the ability to delay gratification in childhood necessary and/or sufficient for better behavioral outcomes 10 years later? Clearly, it is not necessary because people can succeed without delay of gratification, nor is it sufficient because delay of gratification does not guarantee the effect (i.e., it does not always lead to successful performance). Several other factors (e.g., family resources and income) can result in successful performance many years later. Nevertheless, delay of gratification can increase the probability of success for certain groups of individuals and under certain conditions (Mischel et al., 1988, 1989; Casey et al., 2011), especially if it means enhanced perseverance and associated deliberate practice.

Any researcher knows that it would be imprudent to causally ascribe today’s performance to an experimentally exhibited behavior 10 years earlier, because there are numerous intervening variables in the span of 10 years that could easily affect today’s outcomes. In fact, it would be remarkable if the correlations between one variable measured 10 years ago and certain indicators of success today would not be low. These low correlations, however, do not constitute replication failures as such and do not therefore give a license for researchers to throw out the baby with the proverbial bath water (Watts et al., 2018). Low correlations and small effects can be important and meaningful in shedding light on underlying phenomena (Prentice and Miller, 1992). In other words, does anybody seriously think that delay of gratification, or perseverance more generally, is not important for successful human performance? If he/she does, he/she then needs to explain why more than 10,000 h of “deliberate practice” is required for becoming an exceptional performer (Ericsson et al., 1993).

Research on delay of gratification illustrates the difficulty of being able to draw hard empirical conclusions about psychological phenomena and their truth-value. For one thing, seldom or few psychological phenomena are stand-alone effects. Rather, they exert their influence through other factors, which then makes the effects more variable and less replicable, but not less important. For another, because all psychological effects are time-bound and context-bound, their causes are not exclusively either necessary or sufficient as such. For example, although anxiety is not necessary for “choking” (impaired performance), it can be sufficient under many conditions (i.e., in certain context and time). But establishment of sufficient causation between **X** and **Y** with longitudinal data (e.g., research on delay of gratification) poses major methodological and statistical challenges for replications of the original findings.

Rather than being surprised or dismayed by so-called replication failures due to the complexity of psychological effects, they should be embraced not as the final arbiters of scientific truth (no yes-or-no determinations) but merely as additional feedback in the ongoing theoretical and empirical enquiry of multifaceted psychological phenomena. Replication failures can be useful for revealing phenomena’s boundary conditions and informing researchers on the interaction effects of context and time, and individual differences.

More generally, replication failures can aid in developing theories that last for the longest amounts of time over the greatest ranges. Parsimonious theories are such theories (e.g., Newton’s theory) and can be achieved when unimportant or non-influencing variables, overlapping constructs, and exogenous causes are removed, when auxiliary assumptions linked to theory (Earp and Trafimow, 2015) and specified theoretical effects (Witte and Zender, 2018) are tested and replicated, as well as when the relationships are properly theorized instead of adding more variables to an ever-growing list of moderators and mediators. Evidence indicates that “complex models are much less likely to be true than simple models” (Saylors and Trafimow, 2020).

## Replication and Psychological Phenomena

Understanding the role of replication in the scientific process is rooted in the assumption that the identical method has to produce identical results. As argued, this assumption is false because methods themselves are psychometrically limited and because they are not identical from one study to another. In addition, as psychological phenomena exist in different forms, degrees, and contexts, they necessitate employment of the same method in different situations and different methods in the same situation. Failures to conduct replications under this principle have led to unjustified conclusions and assertions, as well as denials of many classic phenomena. Sweeping generalizations have been propagated from the attempted yes-no replications, as was the case with the BBC television prison study claiming to replicate Zimbardo’s Stanford Prison Experiment. The replication bore little resemblance to the original in the employed experimental protocol, not to mention any consideration of other studies on social power. Focusing on Zimbardo’s method, questionable or not, is losing sight of the main thing, the phenomenon itself: how social power affects human behavior.

Regardless of disagreements on the methodological procedures, Zimbardo’s and Milgram’s experiments undeniably showed the power of social influence on individual emotions, thoughts, and behaviors in the reported experimental situations. Even when considering the special lab conditions created in these experiments, no replication can deny the fact that Milgram was able to create an experimental situation in which social power made participants ostensibly hurt other humans. In other words, the obedience effect was observed in that specific situation at that specific time, and it therefore existed then and exists today, even if in a different form and degree.

These original findings do not mean that social situations overpower every individual at all times, as shown by Milgram (1963) himself. While 65% of participants in his experiment gave the highest level of electric shock to the confederate, 35% did not. This demonstrates the essence of psychological phenomena and their empirical verification: studies may discover patterns and replications may confirm them, but patterns, by definition, do not cut across time, situations, and persons. They vary not just between individuals but also within individuals. The problem with patterns is that they are quantitative and statistical patterns from which the operation of psychological effects is inferred. Increased statistical power and larger sample sizes certainly increase the credibility of patterns, but they do not eliminate the “inferential reproducibility” problem (Goodman et al., 2016) that different researchers tend to draw different conclusions from the same data.

There are numerous examples in the literature of methodological liberties replication researchers have taken in attempts to disprove original findings (Doyen et al., 2012; Gerber et al., 2016). The “replicator degrees of freedom” have been shown to lead to unwarranted claims of replication failures (Ramscar, 2016; Bryan et al., 2019). Thus, it cannot be assumed that so-called independent replications are unbiased. In fact, it has been suggested that there is an incentive to find the null result refuting the original finding (Bryan et al., 2019). Refutations can readily be obtained by liberal uses of replicator degrees of freedom [e.g., prior selection of experimental designs and analyses (Bryan et al., 2019; Sherman and Rivers, in press)]. Furthermore, people’s general sensitivity to social influences (Bavel et al., 2016) makes it relatively easy to conduct, unwittingly or not, replication experiments to produce refuting evidence. One way to safeguard against methodological biases would be for replication studies to provide well-developed theoretical rationales that would specify beforehand under what conditions certain effects are likely or unlikely to be found and replicated. Such theoretically based replications would make greater contributions than mere methodological replications.

Regarding the classical studies of psychology, it should not be forgotten that they were original discoveries or demonstrations of various phenomena, which naturally is more important than any replications of them. If Festinger (1957) did not discover cognitive dissonance, there would be no cognitive dissonance to be replicated. Thus, initial theorizing is critical for the advancement of science, and innovation should be promoted and not suffocated by the overemphasis on replication, as Lancet’s editorial (2017) expressed it: “Prescriptive regulation of scientific thought and processes that stifle creativity under a guise of enforcing reliability could ultimately impede discovery and advancement.” This argues for a balance between theoretical innovation and empirical research, including phenomenon and constructive replications investigating potential confounds and testing competing explanations and specified theoretical effects for the generalizability of the original findings (Witte and Zender, 2018).

## Psychology as Science

Although so-called replication failures have raised questions about psychology as a serious science, good news is that in the long run, the value of scientific psychology cannot be diminished by any of it, for one thing, because psychology is the only science that can answer some important questions about human life. For example, why is it that most people do not exercise regularly even though physiological evidence has compellingly demonstrated that “exercise is medicine” for both prevention and treatment of major illnesses? The question cannot be answered by physics, chemistry, biology, engineering, or any other field of science, except psychology, because the answer lies in the operations of the human mind and the brain (Iso-Ahola, 2013, 2018). Similarly, it is the psychological scientists who are tackling the hardest problem of all problems in all of science (Gleiser, 2014): human consciousness. It does not matter even if knowledge is incomplete at the time when answers are provided. Much is known about antecedents of depression and how to treat its effects today, but more will be known tomorrow.

Even though psychological science does not seek to make precise predictions for individual behaviors and concomitant precise replications of them, this does not undermine its scientific status. Instead of precise predictions, psychological science aims to establish patterns and regularities to elucidate general human tendencies, and robust patterns likely explain recent replication successes (Klein et al., 2014; Camerer et al., 2018). However, patterns are just that, patterns, for which there are exceptions that make individual predictions imprecise. Even though specific behavioral predictions cannot be made from general human tendencies, knowing an increasing number of individuals’ general tendencies can enhance the predictive power at the individual level. Despite the fact that “causality operates on single instances, not on populations whose members vary” (Cohen, 1994), statements regarding probabilistic causation can be made at the group level (Pearl and Mackenzie, 2019).

Thus, it is not possible to predict the time, the place, and the name of a particular individual to be involved in the next mass shooting, but factors influencing the probability of the phenomenon’s occurrence can be determined. Even if the resultant information provides explanations in hindsight, cumulating knowledge can become increasingly useful in real life. Difficulties of predicting specific human acts and behaviors highlight why psychology is a complex and “hard” science.

However, inability to make precise predictions for specific behaviors and specific individuals’ behaviors does not diminish the value of psychology as science, certainly no more than a recent failure of the Phillips Curve has been used to question economics as science. The Phillips Curve (i.e., a negative correlation between unemployment and inflation) has served as the basic tenet of economics for over 60 years and guided Federal Reserve in its policy decisions. Yet, the interactive relationships between wages, employment, and inflation are more complicated than any two-variable correlation. Similarly, an explanation of human behavior cannot be reduced to a psychological equivalence of the Phillips Curve. Complexity of human behavior calls for multi-variable theories to explicate stability and variability in cognition, affect, and behavior.

## Role of Theory

Although science can be seen as a dynamic dance between theory, methods, and data (Boker and Martin, 2018), it is important to acknowledge that replications take a backseat to the most important work in the scientific process of discovering truth: theory-building, theory-elaboration of the latent structure of psychological phenomena, model generation, and “continuous model expansion” (Meehl, 1990; Gelman and Shalizi, 2013; Edelsbrunner and Dablander, 2020). Many believe that “scientific progress in psychology will likely depend upon the development of integrated models” (Grice, 2014, p. 22). Recent evidence, however, raises a note of caution about this process as complex models are less likely to true. Saylors and Trafimow’s data (2020) showed that as the number of variables in the model increased, the probability that the model is true decreased rapidly.

Unquestionably, reality is complex if one counts all the hundreds of variables that could affect human affect, cognition, and behavior. In everyday life, though, people do not deliberate over 50 possible reasons why they should or should not go for a walk or run. Instead, they, at least regular exercisers, have delegated decision-making to cognitively less demanding operations of the non-conscious mind (Iso-Ahola, 2018), and this process should therefore be taken into account in theory-building. Sufficiently specified theory would indicate major variables or conditions under which effects could occur.

In general, scientists’ task is to develop theories to explain how the universe functions and how the human mind operates, and for this, empirical feedback is needed. Empirical data can help clarify theories and contribute to the expansion of theoretical models, but they cannot turn psychological phenomena into yes-or-no particles whose existence is determined by replications using arbitrary statistical criteria. Once a clear theoretical, logical, or mathematical case has been made for a phenomenon, be it gravity or cognitive dissonance, the phenomenon will not cease to exist as a deductive truth. Where would physics (and the world) be today without Einstein’s theories? The Higgs boson particle was theorized (logically and mathematically) to exist in 1964 but not empirically verified until 2012. Did the particle not exist in the meantime?

Naturally, false, or even absurd, theories will be proposed from time to time, but they will quickly be dismissed when it is seen that they cannot stand logical, theoretical, or mathematical scrutiny. Granted, in some cases, such dismissals can be premature if theories have not been fully or sufficiently developed. This was evident when the initial idea of quantum mechanics (i.e., quantum entanglement of pairs of particles) was rejected by many physicists, including Einstein who called the entanglement “spooky action at a distance.” Although he and his associates (Einstein et al., 1935) concluded that the quantum mechanics account of physical reality is incomplete, they left the door open by suggesting that “such a theory is possible.” A critical development occurred when Bell published his mathematical “inequality” theorem in 1964, which enabled empirical testing of the two accounts of physical reality (quantum mechanics and Einstein’s view). Bell’s proposal inspired experimental work, but it took more than 50 years before the quantum mechanics explanation was confirmed beyond any reasonable doubt (Hensen et al., 2015; Yin et al., 2017).

There is an important lesson here for psychological science. Theories should not prematurely be dismissed, especially by single replication experiments or even meta-analyses, as empirical verification is a long-term process. Furthermore, given the complexity of psychological phenomena not as yes-or-no particles, empirical evidence is unlikely to be able to deal the final blow to a theory (Popper, 1959; Lakatos, 1976). In light of methodological difficulties of empirical verification, and the decades the process can take, it is illogical that single replication failures of many psychological effects have recently been accorded much more weight than numerous successful replications of the original (Doyen et al., 2012). Since single, direct replications (method replications), even if preregistered and involving many labs, are not any purer methodological demonstrations than the original, they cannot be final arbiters of various phenomena’s existence or non-existence. If, however, replications are based on a diversity of methods and methodological improvements (phenomenon-focused replications), they will be useful for creating new knowledge. It should also be noted that failed replications can help advance science when they lead to revisions and reinventions of the original theory or hypothesis, as in the case of Inzlicht and Schmeichel’s (2012) re-theorizing ego-depletion as a motivational mechanism rather than a resource depletion.

Theories are continually being refined, qualified, expanded, and their boundary parameters being established by empirical tests, as no theory is complete, especially in psychological science where time and context interactively cause considerable variation in behavior. Along with refinement and expansion, the basic idea of a well-developed theory provides the best provisional and propositional explanation for the nature of the universe or the operations of the human mind at the present time. Thus, even when its boundary conditions become better refined and established, the basic idea of cognitive dissonance will not disappear from the general psychological explanation of human behavior. In a similar vein, Newton’s conception of gravity stands even after Einstein took Newton’s theory to a different level by specifying the mechanism to explain gravity in space-time. The upshot is that in a bigger picture, theory and data are interconnected: “Data without theory is lame; theory without data is blind” (Gleiser, 2014). However, the recent elevation of replication into a special status in empirical examination has mistakenly led many to believe that scientific truth is a matter of the pinpoint statistical verification through replication. As argued, such a conclusion is groundless.

## Conclusion

Scientific knowledge is temporary, dynamic, conditional, and relative to contexts in which an examined behavior occurs. As the scientific explanation is always provisional and propositional, no absolute truth exists, either in physics or psychology. Strictly speaking, as the physicist Ethan Siegel concluded, “scientific truth is a myth.”

Time and context are inextricably interwoven in human behavior, with time creating different contexts and contexts creating different experiences of time. The resultant variability poses insurmountable problems for precise replications. Since exact replications are not possible, a replication study is just another empirical investigation in ongoing efforts to establish scientific truth. It does so by refining, qualifying, and expanding an earlier finding, but it cannot declare whether something exists permanently, or not at all. It is therefore more accurate to talk about temporary scientific knowledge than the presence or absence of scientific truth, regardless of claims made by replication researchers. Science builds knowledge incrementally and cumulatively and cannot therefore make categorical pronouncements on the existence or non-existence of a given truth by means of replications, especially single replications; nor can scientific truth be defined as statistical truth.

Psychological phenomena are essentially interactive effects, exerting their influence through many variables as opposed to stand-alone “main” effects. This complexity greatly increases the likelihood of replication failures. Moreover, because psychological phenomena are time- and context-bound, their causes are rarely exclusively either necessary or sufficient, and never both. This complex nature of causality of psychological effects creates major problems for demonstrations of the replicability of previous findings. While external (physical) conditions can be made to approximate those of the original study, however, internal conditions between the original and replication studies are never the same. That is, there is not a single report in the literature that would have shown a similar (much less identical) degree of both conscious and non-conscious processing by participants of the original and replication studies. In short, unless it is known what is in the minds of replication study participants, one cannot be certain if the original study is being replicated.

The fact that exact replications are not possible does not mean that nothing can be known. Science is still “the best game in town” for creating new basic and applied knowledge for better understanding the universe and the human mind (Kerlinger, 1973). Even though scientific knowledge is limited and incomplete, it does not mean that psychological phenomena do not exist. Their existence, however, is not a matter of the pinpoint statistical verification through replication but primarily a function of a continual dynamic dance between theory, method, and data. In this process, phenomenon and constructive replications play an important role as long as they are continuous and iterative, avoiding declarations of the “basic existence” of phenomena and exercising constrains in making generalizations. Replications become constructive and useful for the advancement of science when they employ the same or similar method in different contexts and different methods in the same or similar contexts of human behavior on one hand, and when they lead to revisions and reformulations of existing hypotheses and theories on the other. In this process, emphasis is placed on phenomenon replication rather than method replication.

## Author Contributions

The author confirms being the sole contributor of this work and has approved it for publication.

## Conflict of Interest

The author declares that the research was conducted in the absence of any commercial or financial relationships that could be construed as a potential conflict of interest.
